# Worsened Financial Situation During the COVID-19 Pandemic Was Associated With Depressive Symptomatology Among University Students in Germany: Results of the COVID-19 International Student Well-Being Study

**DOI:** 10.3389/fpsyt.2021.743158

**Published:** 2021-12-16

**Authors:** Sarah Negash, Nadja Kartschmit, Rafael T. Mikolajczyk, Stefan Watzke, Paula Mayara Matos Fialho, Claudia R. Pischke, Heide Busse, Stefanie M. Helmer, Christiane Stock, Hajo Zeeb, Claus Wendt, Yasemin Niephaus, Andrea Schmidt-Pokrzywniak

**Affiliations:** ^1^Institute for Medical Epidemiology, Biometrics and Informatics, Interdisciplinary Center for Health Sciences, Medical School of the Martin-Luther University Halle-Wittenberg, Halle (Saale), Germany; ^2^Clinic for Psychiatry, Psychotherapy and Psychosomatics, University Hospital Halle-Saale, Halle (Saale), Germany; ^3^Medical Faculty, Institute of Medical Sociology, Centre for Health and Society, Heinrich Heine University Duesseldorf, Duesseldorf, Germany; ^4^Department Prevention and Evaluation, Leibniz-Institute for Prevention Research and Epidemiology – BIPS, Bremen, Germany; ^5^Institute of Health and Nursing Science, Charité—Universitätsmedizin Berlin, Corporate Member of Freie Universität Berlin and Humboldt-Universität zu Berlin, Berlin, Germany; ^6^Health Sciences Bremen, University of Bremen, Bremen, Germany; ^7^Department of Social Sciences, University Siegen, Siegen, Germany

**Keywords:** university students, COVID-19 pandemic, financial situation, depressive symptoms, mental health

## Abstract

**Background:** Previous findings suggest that university students are at an elevated risk to experience financial hardship and to suffer from depressive symptoms. This vulnerability may have substantially increased during the coronavirus disease 19 (COVID-19) pandemic which might have affected students' socio-economic situation but possibly also their mental well-being. We examined whether the financial situation changed during the COVID-19 pandemic among German university students, and whether changes were associated with mental well-being.

**Methods:** We conducted a cross-sectional online survey in May and July 2020 at five German universities. Participants were asked, if they had sufficient financial resources to cover monthly expenses before and during the pandemic. The answer options were dichotomized into worsened and no change/better financial situation compared to before the COVID-19 pandemic. Depressive symptoms were assessed using the CES-D 8 scale. For examining associations between sociodemographic, study-related, and financial factors and “worsened financial situation,” we ran a generalized linear mixed model. To assess associations between depressive symptoms and worsened financial situation, we performed a linear mixed model.

**Results:** We included 7,199 participants in the analyses (69% female, 30% male, 1% diverse, mean age: 24 years, standard deviation: 4.7). Overall, 25% of the participants reported to have a worsened financial situation at the time of the survey than in the time before COVID-19. Factors associated with a worsened financial situation were migration background, parents not being academics, not being able to borrow money, and payment of tuition fee by student and loan [odds ratios (OR) ranging from 1.20 to 2.35]. Factors associated with lower odds were: being single, living with others, studying a health-related field, being enrolled in a doctoral/Ph.D. or state exam program, and publicly funded tuition/tuition paid with a scholarship (OR ranging from 0.42 to 0.80). A worsened financial situation was associated with 1.02 points more on the CES-D 8 scale (95% CI: 0.80–1.24).

**Conclusion:** Our results suggest that the pandemic put a number of students under financial strain with detrimental consequences for their mental well-being. Renewed attention must be paid to this vulnerable group to prevent the potentially damaging effects on their mental health.

## Introduction

The coronavirus disease 19 (COVID-19) emerged in China at the end of 2019 and in March 2020 the World Health Organization (WHO) declared the outbreak a global pandemic (1). The lockdown and restrictive measures introduced in response, aimed at limiting transmission of the virus, affected higher education students worldwide, and concerns were raised regarding the financial implications of the pandemic and the subsequent impact on their mental well-being.

Pre-COVID-19 studies on students' economic situation show that many were already experiencing financial concerns (2, 3). Factors found to be associated with experiencing financial difficulties more often among university students were being female and older, having a migration background and having children, as well as being enrolled in a Bachelor's compared to a Master's degree program (4). Similarly, results of other international, as well as national studies, suggested that relationship status, length of study, and parents' academic background are associated with different indicators of financial circumstances (5, 6). International studies demonstrate that many students worldwide are currently facing financial challenges due to the COVID-19 pandemic (4, 7, 8). Students relying on part-time jobs to finance their livelihood and those that lost their jobs due to the lockdown might experience financial hardship. Students who were financially supported by their parents might not or only partly continue to receive financial support, as in the wake of the pandemic parents might face a worsened income situation themselves (6, 9). Studies suggest that financial insecurity is putting students in a stressful situation and this, in turn, is affecting their mental well-being (2, 10). A recent national survey on the economic situation of university students in Germany, conducted in the time period from January to Mid-June 2020, reported that students often experienced a worsened financial situation during the summer semester 2020 in terms of their earnings when compared to their situation one semester before (6). However, this study did not investigate the potential impact on students' mental well-being caused by this increased financial stress.

University students are confronted with a panoply of stressors, including academic pressure (11), concerns about the future (12), life-stage transitions, and financial worries (13), all of which can promote the occurrence of mental health issues. Thus, they have been identified as a group generally at risk for developing common mental health disorders (14, 15) and they consistently show higher levels of mental health disorders when compared to people of the same age that are not university students and the general population in Germany (16) and elsewhere (17). Previous research indicates that the pandemic exacerbated students' vulnerability, as the measures adopted to fight against it, such as quarantine measures and social distancing, carry a unique stress (18, 19).

A number of international studies reported that financial strain and financial worries are linked to adverse mental health outcomes even before the COVID-19 crisis (2, 4, 15). Jessop et al. (14) found associations between higher levels of financial concern and worsened overall mental well-being in British university undergraduate students. A study among Norwegian college and university students found a social gradient pattern with more favorable mental well-being outcomes for students never experiencing financial difficulties compared to students frequently experiencing financial difficulties (4).

In the context of the SARS pandemic 2002/03, financial stressors and poor mental well-being were found to be linked (20). Currently, it is known from the literature that policies to contain the COVID-19 pandemic led to increased financial and psychological stress in university students (8, 19). Recent evidence from Germany also indicates the importance of financial challenges as a stress factor for university students (6, 21). To date, the factors that may contribute to a worsened financial situation due to the COVID-19 pandemic in university students in Germany have not been investigated. Additionally, while the mental well-being of students during the pandemic has been well-documented (22–25), financial hardship was rarely included when examining mental well-being. Moreover, when included, mainly changes in income were examined in existing studies, representing only one part of the students' financial resources (6, 21). By bridging the abovementioned gaps, this study should make an important contribution to the literature on the association of financial condition and mental well-being among university students in the context of the pandemic.

This study therefore aims (a) to examine the change in the financial situation of German university students during the COVID-19 pandemic, (b) investigate which factors were associated with a worsened financial situation, and (c) assess associations between a worsened financial situation and depressive symptoms in this group, pertaining to the experiences of university students during the period of the first COVID-19 outbreak between January and July 2020.

## Methods

### The COVID-19 International Student Well-Being Study

Data of this study were collected in the context of the COVID-19 International Student Well-Being Study (C19 ISWS). The aim of the C19 ISWS was to examine the consequences of the COVID-19 pandemic on the well-being of students at universities in Europe, North America, and South Africa via an online survey. The multi-center study is led and coordinated by a research team at the University of Antwerp in Belgium. The questionnaire was independently translated by two members of the German study team according to the translation protocol. In addition to students' sociodemographic characteristics, information on different areas of student life were captured in the survey (e.g., financial conditions before and during the COVID-19 outbreak, the current level of mental well-being, and perceived stressors). Further information on the development of the survey can be found elsewhere (26).

### Study Population and Context

For this study, we used data from the German study sites. Five German universities participated in the C19 ISWS (Heinrich Heine University Duesseldorf, University of Siegen, University of Bremen, Martin-Luther-University Halle-Wittenberg, and Charité—University Medicine Berlin). The local Ethics Committees approved the study. Currently enrolled students aged 17 years and older were eligible to take part in the online survey. Via email distribution lists, notifications on, for example the university homepage and social media, the students were invited to participate.

In March 2020, the German government declared drastic restrictions aiming at reducing the number of COVID-19 infections. Universities, including lecture halls, and libraries were closed, as well as cafés, restaurants, and non-essential shops. Additionally, social distancing requirements were announced, including restrictions on how many people could gather in- and out-side. Mid-May and onwards, some restrictions were relaxed; restaurants and non-essential shops were opened again. However, most of the restrictions at the participating universities remained and online classes were predominantly taught. There were few exceptions for in-person classes, for example for some classes of medical students requiring hands-on practice. All universities gathered data during the time most restrictions were relaxed (May 12–29th, 2020 at the Heinrich Heine University Duesseldorf, University of Siegen, University of Bremen and Charité—University Medicine Berlin; July 14–29th, 2020 at the Martin Luther University Halle-Wittenberg).

### Measurements

#### Change in Financial Situation During COVID-19

Change in financial situation was measured with a 5-point Likert scale asking whether the students had sufficient financial resources to cover monthly costs before the COVID-19 pandemic, as well as during the COVID-19 pandemic with the following answer options: (1) strongly agree, (2) agree, (3) neither agree nor disagree, (4) disagree, (5) strongly disagree. “Before the outbreak” referred to the average situation during the month prior to the time point when the first COVID-19 measures were implemented, whereas “currently” referred to the week prior to filling out the questionnaire. The change in financial situation was calculated by subtracting the score before the pandemic from the score representing the pandemic situation and dichotomized into: (1) no change or improvement, and (2) worsened.

#### Depressive Symptoms

We assessed depressive symptoms using the Center for Epidemiological Studies Depression Scale (CES-D 8 scale) (27). The reliability and validity of the scale are confirmed in research among the general population [e.g., (28)], with values for Cronbach's alpha of 0.80 for men and 0.82 for women (29). The scale consists of eight items: feeling depressed, feeling that everything was an effort, restless sleep, feeling sad, feeling lonely, could not get going, as well as feeling happy, and enjoying life. A 4-point Likert scale was used to indicate how often during the past week the above-mentioned feelings occurred: (0) none or almost none of the time, (1) some of the time, (2) most of the time, (3) all or almost all of the time. To derive the CES-D 8 score, we summed up all eight items, reversing the items “feeling happy” and “enjoying life” beforehand. The theoretical range of the score is 0–24, with a lower score indicating less pronounced depressive symptoms.

#### Factors Associated With Worsened Financial Situation and/or Depressive Symptoms

We included the following sociodemographic characteristics: age in years, gender (female, male, diverse), relationship status (single, in a relationship, it is complicated), having a migration background (respondent or at least one parent being born outside of Germany), educational level of parents (at least one parent academic vs. both parents non-academics), and living situation (with parents, student hall, with others, alone, other).

We included the following study-related factors: Field of study (health-related, such as Medicine or Public Health, vs. not health-related, such as natural sciences and humanities), and study program (Bachelor, Master, Doctoral/Ph.D., and state exam).

Eight questions were asked to assess students' perceived academic frustrations due to the pandemic and an index named “academic frustrations” was created based on the following eight questions (30): (1) increased workload, (2) knowing less what is expected in courses, (3) being concerned not to be able to complete the academic year, (4) poorer quality of education, (5) change in teaching methods caused stress, (6) sufficient information about changes by university, (7) satisfaction with measures at university, (8) feeling able to talk with university staff about concerns. A 5-point Likert scale was used with the following options: (1) strongly agree, (2) agree, (3) neither agree nor disagree, (4) disagree, (5) strongly disagree. Items were reversed when applicable and summed up, so that higher scores indicated higher academic frustration. The score has a theoretical range from 8 to 40.

To assess social interactions, we asked whether students have a “person to discuss intimate and personal matters with” (yes/no).

In order to further assess the financial situation of the students, payment of tuition fee (by other person, e.g., parents, grandparents, spouse, by student him/herself, publicly funded and scholarship, loan, and combination of the before mentioned/other) was considered. In Germany, the tuition fee is paid twice a year and includes usually transportation, administrative contributions, and costs for the student union. Moreover, for some students there are additional fees, e.g., for students who already have a degree in a different field. Additionally, to assess student's ability to borrow money, students were asked if they could easily borrow 500 Euros within two days from at least one person (yes/no).

### Statistical Analysis

For all included variables, we presented descriptive statistics using means and standard deviations for metric variables and total numbers and percentages for categorical/dichotomous variables.

For the outcome “worsened financial situation,” we ran a generalized mixed effect model. For the outcome “depressive symptoms,” measured with the CES-D 8, we used a linear mixed effect model. We included study site as random effects in both models. We included the following variables in the regression for the outcome “worsened financial situation”: sociodemographic variables, study-related factors (except perceptions of academic frustrations during the COVID-19 pandemic), and study-related finances. We ran a crude regression model for the outcome “depressive symptoms” and a model adjusted for the following variables: sociodemographic variables, study-related factors, and social interactions. We selected the above-mentioned covariates based on a thorough literature review [e.g., (13, 14, 31, 32)]. In both models, we tested the included variables for multicollinearity, but did not find evidence thereof. Hence, we included all variables simultaneously in the models (variance inflation factor <5) (33).

In addition, we examined a worsened financial situation and the change in hours worked in paid jobs during the pandemic descriptively. Finally, we examined the change in hours worked in paid jobs in different subgroups (health-related field of study, payment of tuition fee, and study program).

All analyses were conducted using the statistical software R (34).

## Results

### Sample Characteristics

A total of 8,725 participants completed the survey. After excluding participants with missing values for any of the included variables (except information on hours spent in paid jobs, which was examined in an additional analyses leaving 2,906 participants who either worked before and/or during the pandemic after excluding missing and implausible information), 7,199 observations were left for analysis (82%). In the sample, 69% were female, 30% male, and 1% diverse with a mean age of 24 years (*SD* = 4.7). Twenty-five percent of the participants reported to have a worsened financial situation during the pandemic compared to before the pandemic. The mean CES-D 8 score was 9.2 (*SD*: 4.7). A more detailed description of the sample is given in Table 1.

**Table 1:** Participants' characteristics (*n* = 7,199).

Socio-demographic	Age, mean (SD)	24.1 (4.7)
	Female, *n* (%)	4,977 (69.1)
	Male, *n* (%)	2,144 (29.8)
	Diverse, *n* (%)	78 (1.1)
	Single, *n* (%)	3,040 (42.2)
	In a relationship, *n* (%)	3,870 (53.8)
	It is complicated, *n* (%)	289 (4.0)
	Migration background, *n* (%)	1,676 (23.3)
	Both parents not academics, *n* (%)	1,710 (23.8)
	Living with parents, *n* (%)	2,312 (32.1)
	Living in student hall, *n* (%)	381 (5.3)
	Living in accommodation with others, *n* (%)	3,255 (45.2)
	Living alone, *n* (%)	1,099 (15.3)
	Other form of accommodation, *n* (%)	152 (2.1)
Study related	Health related field of study, *n* (%)	1,610 (22.4)
	Perceptions of academic frustrations during COVID-19 pandemic, mean (SD)^*^	24.0 (5.7)
	Bachelor, *n* (%)	3,485 (48.4)
	Master, *n* (%)	1,527 (21.2)
	Doctoral, Ph.D., *n* (%)	327 (4.5)
	State exam (e.g., law, medicine), *n* (%)	1,735 (24.1)
	Other (e.g., Diploma), *n* (%)	125 (1.7)
Finances	Tuition fee publicly funded, scholarship, *n* (%)	1,093 (15.2)
	Tuition fee paid by other person, *n* (%)	2,665 (37.2)
	Tuition fee paid by student, *n* (%)	2,269 (31.5)
	Tuition fee paid with loan, *n* (%)	197 (2.7)
	Combination of above or other, *n* (%)	975 (13.5)
	Not being able to borrow money from at least one person, *n* (%)	397 (5.5)
	No change/better financial situation, *n* (%)	5,391 (74.9)
	Worse financial situation, *n* (%)	1,808 (25.1)
Depressive symptoms	CES-D 8 score, mean (SD)^**^	9.2 (4.7)
Social contact	No person to discuss intimate and personal matters with, *n* (%)	636 (8.8)
Study site	Charité—University Medicine Berlin, *n* (%)	713 (9.9)
	University Bremen, *n* (%)	1,813 (25.2)
	Heinrich-Heine University, *n* (%)	592 (8.2)
	University Siegen, *n* (%)	1,663 (23.1)
	Martin-Luther-University Halle-Wittenberg, *n* (%)	2,369 (32.9)
	Other, *n* (%)	49 (0.7)

* *Range 8–40, higher scores indicating lower satisfaction*.

** *Range 0–24, higher scores indicating higher frequency of depressive complaints*.

**Figure 1:**
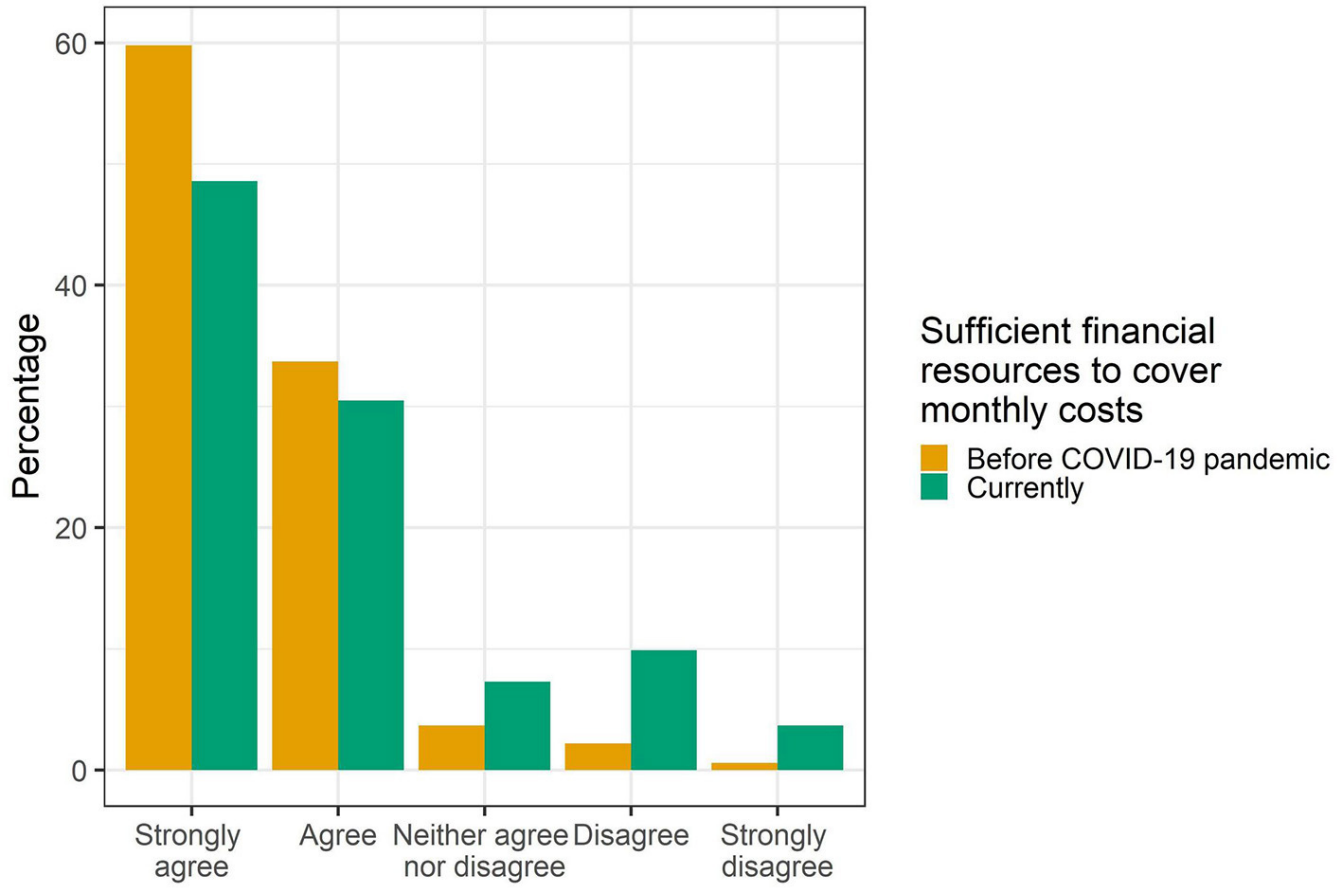
Perceived sufficiency of financial resources among university students (*n* = 7,199).

Approximately 60% of the participants strongly agreed that they had sufficient financial resources to cover monthly costs before the pandemic, while less than half strongly agreed with this statement during the COVID-19 pandemic. Likewise, the percentage of students strongly disagreeing and disagreeing with this statement was higher for the pandemic situation compared to before the COVID-19 pandemic (Figure 1).

**Figure 2:**
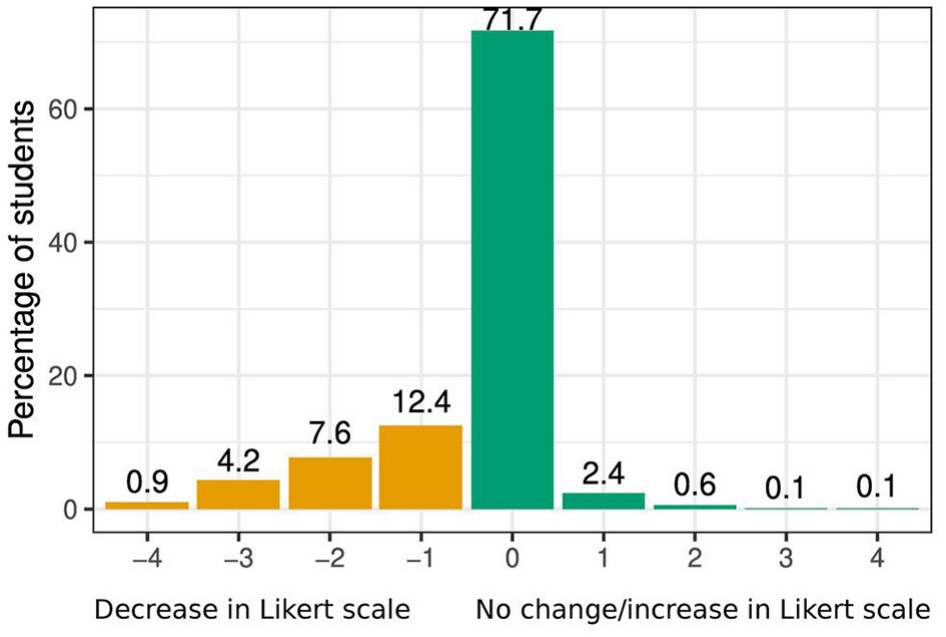
Change in financial resources before and during the COVID-19 pandemic (*n* = 7,199).

Of the 25% of the study population that indicated a decrease in financial resources to cover monthly costs, the majority indicated a 1-point decrease on the 5-point Likert scale. Few reported a 4-point decrease (from strongly agree to strongly disagree). Only few reported an increase and the majority of them reported a 1-point increase. However, 72% reported no change in financial resources (Figure 2).

### Characteristics Associated With Worsened Financial Situation

In the generalized mixed effect model, factors associated with worsened financial situation were migration background, both parents not being academics and not being able to borrow money from at least one person. Students who paid tuition fee themselves, with a loan, and a combination had higher odds for having a worsened financial situation vs. tuition fee paid by another person. Factors associated with lower odds for having a worsened financial situation were being single, living with parents and in an accommodation with others vs. living alone, studying a health-related field, being enrolled in Doctoral/Ph.D. and state exam program vs. Bachelor program, and publicly funded tuition/tuition fee paid with scholarship vs. tuition fee paid by another person. Gender, having a complicated relationship status, and being enrolled in Master's program vs. Bachelor program were not associated with a worsened financial situation (Table 2).

**Table 2:** Factors associated with worsened financial situation^*^ (*n* = 7,199).

		**Odds ratio (95% confidence interval)**	No change/better financial situation, *n* (%)^**^	Worsened financial situation, *n* (%)^**^
Socio-demographic characteristics	Age, per year	1.01 (1.00–1.03)	24.0 (4.8)	24.5 (4.5)
	**Gender**			
	Female	Reference	3,714 (74.6)	1,263 (25.4)
	Male	0.90 (0.79–1.01)	1,624 (75.7)	520 (24.3)
	Diverse	1.10 (0.67–1.82)	53 (67.9)	25 (32.1)
	**Relationship status**			
	In a relationship	Reference	2,845 (73.5)	1,025 (26.5)
	Single	0.75 (0.67–0.85)	2,339 (76.9)	701 (23.1)
	It is complicated	1.02 (0.78–1.35)	207 (71.6)	82 (28.4)
	**Migration background**			
	No migration background	Reference	4,256 (77.1)	1,267 (22.9)
	Migration background	1.58 (1.39–1.79)	1,135 (67.7)	541 (32.3)
	**Education of parents**			
	At least one parent with academic education	Reference	4,189 (76.3)	1,300 (23.7)
	Both parents not academics	1.20 (1.06–1.37)	1,202 (70.3)	508 (29.7)
	**Living situation**			
	Living alone	Reference	790 (71.9)	309 (28.1)
	Living with parents	0.80 (0.67–0.95)	1,744 (75.4)	568 (24.6)
	Living in student hall	0.78 (0.59–1.03)	292 (76.6)	89 (23.4)
	Living in accommodation with others	0.74 (0.63–0.87)	2,461 (75.6)	794 (24.4)
	Other form of accommodation	0.88 (0.60–1.30)	104 (68.4)	48 (31.6)
	**Field of study**			
	Not health related field of study	Reference	4,021 (71.9)	1,568 (28.1)
Study-related characteristics	Health related field of study	0.54 (0.46–0.65)	1,370 (85.1)	240 (14.9)
	**Study program**			
	Bachelor	Reference	2,472 (70.9)	1,013 (29.1)
	Master	0.89 (0.77–1.03)	1,110 (72.7)	417 (27.3)
	Doctoral, Ph.D.	0.42 (0.29–0.59)	286 (87.5)	41 (12.5)
	State exam (e.g., law, medicine)	0.73 (0.61–0.86)	1,432 (82.5)	303 (17.5)
	Other (e.g., Diploma)	1.05 (0.69–1.58)	91 (72.8)	34 (27.2)
	**Payment of tuition fee**			
	Tuition fee paid by other person	Reference	2073 (77.8)	592 (22.2)
Financial indicators	Tuition fee publicly funded, scholarship	0.79 (0.66–0.95)	884 (80.9)	209 (19.1)
	Tuition fee paid by student	1.20 (1.04–1.38)	1,639 (72.2)	630 (27.8)
	Tuition fee paid with loan	1.78 (1.30–2.43)	117 (59.3)	80 (40.6)
	Combination of above or other	1.42 (1.24–1.75)	678 (69.5)	297 (30.5)
	**Ability to borrow money from at least one person**			
	Yes	Reference	5,183 (76.2)	1,619 (23.8)
	No	2.35 (1.90–2.91)	208 (52.4)	189 (47.6)

* *Study site included as random effect in model, reference category: no change/better financial situation*.

** *For age mean and standard deviation are presented*.

### Association Between Worsened Financial Situation and Depressive Symptoms

Worsened financial situation was associated with a 2-point increase in CES-D 8 score in the unadjusted model. After adjustment, the decrease was about one point (Table 3).

**Table 3:** Association between worsened financial situation^*^ and CES-D 8 score (*n* = 7,199).

	**β**	**(95% Confidence interval)**
Worsened financial situation (crude)	2.13	(1.89–2.37)
Worsened financial situation^**^	1.02	(0.80–1.24)

* *Reference category: no change/better financial situation*.

** *Adjusted for age, gender, relationship status, migration background, educational level of parents (at least one academic vs. both no academics), living situation, field of study (health vs. other), perception of academic frustrations, study program, and no person to discuss intimate and personal matters with. Study site was included as random effect*.

### Change in Working Hours in Paid Jobs and Worsened Financial Situation

Of the 5,443 persons who provided information on hours spent in paid jobs, 2,537 reported to neither have worked before nor during the pandemic and 2,906 have worked either before or during the pandemic.

**Figure 3:**
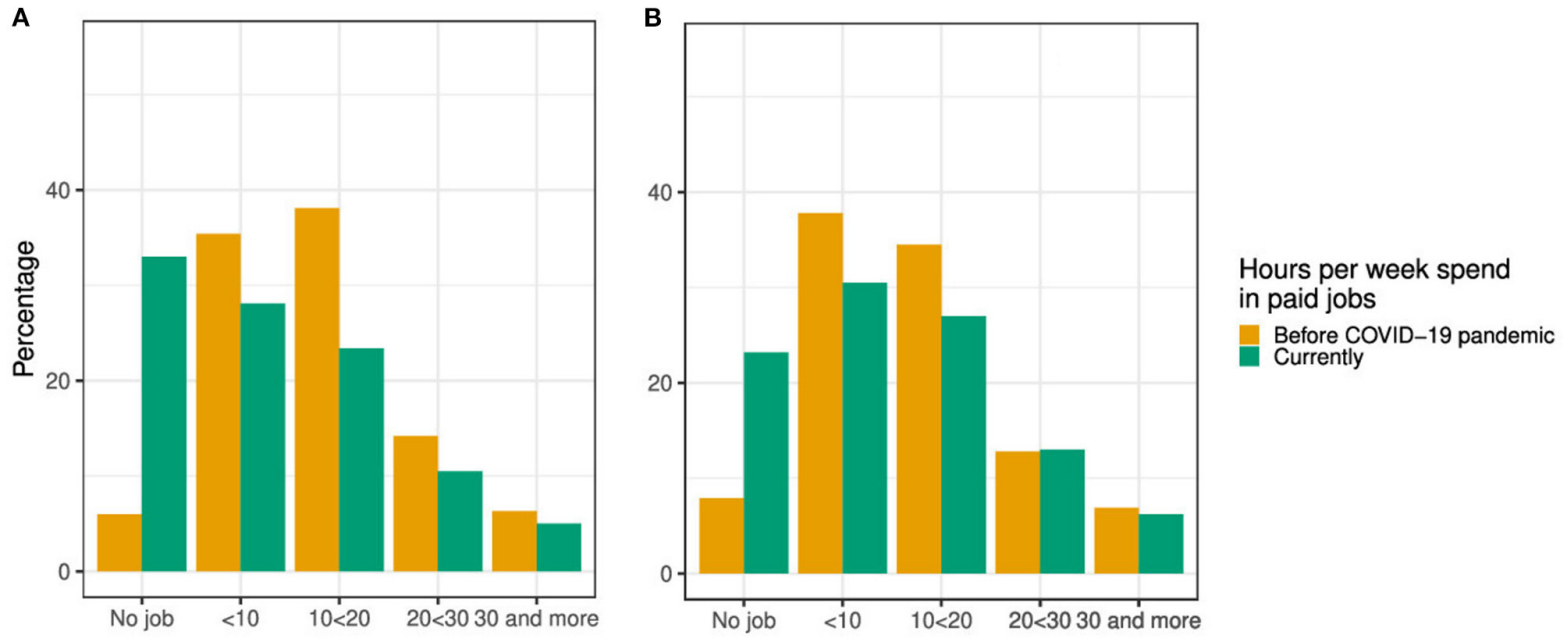
Hours per week spend in paid jobs before the COVID-19 pandemic vs. during pandemic for **(A)** students reporting a worsened financial situation and **(B)** students reporting no change/better financial situation during pandemic (*n* = 2,906).

Of the 2,906 students that worked before or during the pandemic, a higher percentage of students with worsened financial situation reported to have currently no job when compared to students with no change/better financial situation (Figure 3).

Of the 2,906 students, about 80% of the students who reported no change or an increase in working hours, respectively, reported no change/better financial situation. About half of the students who reported a decrease in working hours reported a worsened financial situation (Table 4).

**Table 4:** Change in hours spend in paid jobs and change in financial situation (*n* = 2,906).

	**No change/better financial situation, *n* (%)**	**Worsened financial situation, *n* (%)**
No change in hours worked in paid jobs	1,057 (81.7)	236 (18.3)
Increase in hours worked in paid jobs	289 (82.6)	61 (17.4)
Decrease in hours worked in paid jobs	608 (48.1)	655 (51.9)

**Figure 4:**
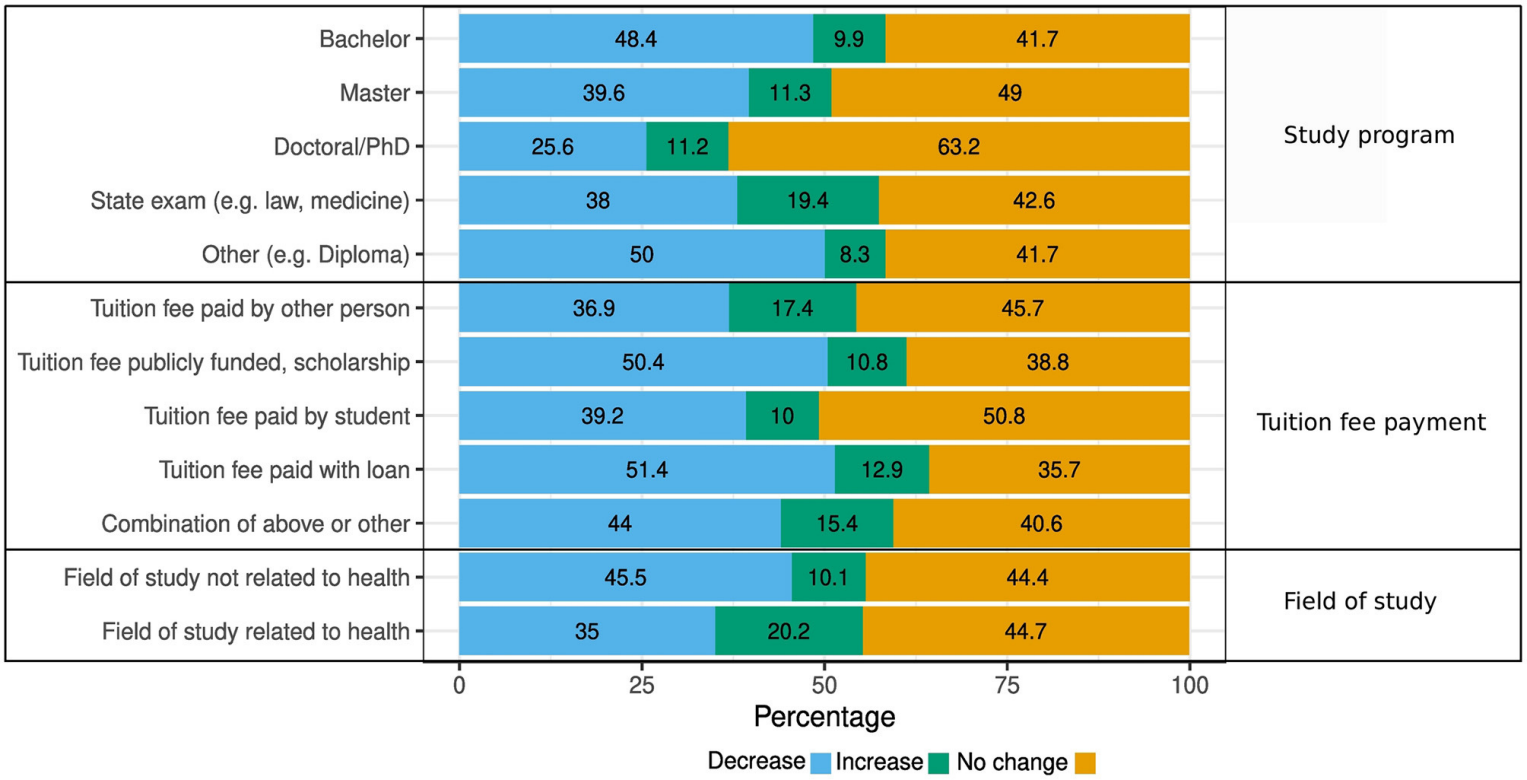
Change in hours worked in paid jobs stratified by study program, payment of tuition fee, and field of study (*n* = 2,906).

When examining the change in hours worked in paid jobs in students who either worked before or during the pandemic, a higher percentage of students studying in the state exam program (e.g., law, medicine) reported an increase in working hours when compared to the other study programs. This is likely because people studying medicine are included in this group and were more likely to work more than during the pandemic than before due to new positions offered (e.g., in health care facilities or health departments). This is also seen when comparing people studying a health-related field vs. a field not related to health. Half of the students who paid their tuition fee with loan reported a decrease in working hours, as well as students with publicly funded tuition fee/tuition fee paid with scholarship (Figure 4).

## Discussion

The present study investigated factors associated with a worsened financial situation and the association between a worsened financial situation and depressive symptoms in German university students during the period of the first restrictions due to the COVID-19 pandemic. A quarter of the students experienced a worsened financial situation during pandemic. Factors associated with a worsened financial situation were, for example, having a migration background, parents not being academics, and payment of tuition fee by student and loan. Our findings suggest that a worsened financial situation was associated with depressive symptoms.

### Changes in Students' Financial Situation During COVID-19

The pandemic caused substantial economic harm resulting in students losing their part-time jobs, which is potentially endangering their livelihood. During the pandemic, less than half of the students strongly felt they had sufficient financial resources, markedly less than prior to the pandemic (60%). These results corroborate the findings of Becker and Lörz (6) who found that for almost 40% of the working students in Germany, the employment situation worsened during the pandemic, either due to job loss, unpaid leave or a reduction in working hours. Similarly, our results indicate that the financial situation could have worsened due to the decrease in working hours. Specifically, students enrolled in a bachelor's degree program or other (e.g., diploma), students who paid their tuition fee with loan or had them publicly funded, and those studying a field not related to health reported a decrease in working hours. Kohls et al. (21) also showed that income decreased for 16% of the students during the pandemic.

Whereas, previous published studies only focused on students' employment situation (6, 21), this study investigated a wider range of financial resources for students. In fact, the means of paying the tuition fee also played a role, whereby those students who paid them for example with a loan and those who paid the fee themselves were more likely to experience a worsened financial situation, as were those who reported no access to *ad hoc* financial support. These predictors suggest a restricted financial flexibility and stability for some students, making an association with an unfavorable financial situation plausible. Furthermore, our study revealed that being single and enrolled in a Doctoral/Ph.D. and state exam program, studying a health-related field, and having their tuition fee publicly funded were found to be protective of having a worsened financial situation. In Germany, many doctoral students work as research associates, and thus, had no change in income during the pandemic. Similarly, students studying a health-related field often have side jobs in the health care system that they most likely did not lose during the pandemic, maintaining a stable financial situation.

Several factors were identified as predictors for experiencing a worsened financial situation, such as the sociodemographic factors older age, having a migration background and both parents not being academics. These findings converge with prior research on financial difficulties of German students (6), indicating financial hardship for some student groups in particular. Berkes et al. (35) suggested that social inequalities among German students may increase during the pandemic, as those students from non-academic families more frequently rely on their employment to finance their livelihood, compared to those from academic families (54 vs. 44%). At the same time, non-academics were more affected by short-time work and job loss during the pandemic which, in turn, may affect the financial support for their children (6).

In line with findings of Okruszek et al. (36) who found loneliness to predict higher financial problems risk in Polish young adults, we found that living with others to be associated with lower odds of having a worsened financial situation. Although living alone does not imply someone feeling lonely, living with others could contribute to feeling less lonely. Students living alone might know less about possibilities to receive financial support or may experience less financial support from close friends or family members, as they may not communicate their financial worries with their social network. In particular, in times of the COVID-19 pandemic, loneliness was reported to be associated with increased levels of depressive symptom (37, 38), and this, in turn, with financial difficulties (21).

### Relationship Between Worsened Financial Situation and Mental Well-Being

Our findings indicate that a worsened financial situation for students was associated with a higher number of depressive symptoms, also after adjustment. This is in line with findings from previous research (2, 21, 39). For example, a study among medical and psychology students in Germany demonstrated that students with financial worries had a higher depression score than their counterparts without financial worries (40). This finding is also supported by Rabkow et al. (41) who identified financial burden as one of the key risk factors for depressive symptoms in German law students. Similarly, a study assessing financial difficulties and student health among Norwegian college and university students found that students often experiencing financial difficulties reported more mental health problems, including depression compared to those never experiencing financial difficulties (4). Further, Andrews and Wilding indicate in a longitudinal study from 2004 that financial strains had a significant impact on the manifestation of anxiety and depressive symptoms among British university students (39).

The mean score of the CES-D 8 scale of German university students in the present study exceeded those recently published of the general adult German population (9.2 *SD* = 4.7 vs. 5.6 *SD* = 4.1), reported in a representative study during a non-pandemic setting (28). Prior literature has suggested that students had higher levels of depressive symptoms than the general population (27). The stressors that are faced by university students in the first place are different from those of the general population or that of their peers, including fulfilling academic demands, establishing new social networks in new environments, and financial pressures (39).

Our results provide first insights into the impact of the COVID-19 pandemic in a large sample of German university students, and more specifically on their financial situation and the impact on their mental well-being. Another strength is that various factors were assessed that were possibly associated with a worsened financial situation, including information about who paid the tuition fee. Some limitations of our study are also worth noting when interpreting the results. All data were collected during the first wave of the COVID-19 pandemic, however, at two different time points (four universities in May and one in July 2020). During both time points implemented measures were comparable and all five universities had switched for the most part to online learning. The short version of the CES-D (CES-D 8) was used to assess mental well-being which is a validated instrument for assessing mental well-being. It should be noted, though, that findings based on CES-D 8 do not reflect a clinical diagnosis. Due to the convenience sample in this study, the generalizability of our results is limited. Voluntary participation could have led to selection bias, with students more affected by the pandemic possibly overrepresented in a survey addressing this issue.

Pre-existing mental health conditions were not assessed in our study and the cross-sectional design of the study does not allow causal interpretation of the results. Self-report measures were used to assess sufficiency of financial resources which may have led to under- or over-reporting of the students' financial situation. Participants, however, were made aware of the anonymity of the survey.

## Future Research and Implications

Longitudinal studies are necessary to investigate the impact of a changed financial situation during the COVID-19 pandemic on students' mental well-being in the long-run. Given the fact that our reports were retrieved during the period of the first COVID-19 pandemic outbreak in Germany, it would be of interest to see whether those students whose financial situation has worsened during that time, managed to stabilize their financial situation during the further course of the pandemic.

The results of this study indicate that there may be a need to increase financial support to students. In fact, to a certain extent, this happened later during the pandemic through dedicated financial instruments. The financial aid that was provided by the German educational policy consisted of three components: funding under the Federal Training Assistance Act (BAföG); financial aid, allocated by student services organizations; as well as temporary, interest-free student loans from the German state owned Credit Institute for Reconstruction (KFW) (6). In addition, universities may support students by offering affordable housing, as studies identify housing insecurity as a problem for students impacting their well-being (42, 43), which exacerbated during the pandemic (44, 45). At the individual level, financial education has been suggested to be beneficial for students, as it increases their awareness for available services and resources, while reducing tendencies of overestimation of one's own level of competency (46). Seeking professional financial help may reduce the financial burden for students and subsequently may have a positive impact on their mental well-being. Future research is warranted to determine the utilization of existing services that are in place to assist university students with their finances, and further unveil barriers that hinder them to access those.

Future research is also necessary to unveil effects of the pandemic on students' mental health in later phases of the pandemic. As has been observed in the case of health care workers in the aftermath of the COVID-19 outbreak, there may be a possibility that the effects of the pandemic on students may continue beyond the peak of the pandemic itself (47). Qualitative research in the form of focus groups and interviews should be conducted, in order to identify, on the one hand, pandemic-related stressors, and, on the other hand, support options named by those affected themselves (48).

A bottom-up methodology is being suggested in the development of preventive measures that address the mental well-being of university students to assure reaching the target population's needs (49). Interventions aimed at improving stress management are suggested to primarily be implemented for enhancing students' health (48).

Students have been shown to have poorer mental health outcomes as a result of the pandemic (21). Whilst the present study investigated the depressive symptoms of students, it would also be important to assess the incidence of physician-based diagnosis of depression in students during the COVID-19 pandemic, which would also require therapeutic actions. Interventions that train and ultimately enable students to deal with psychological stress should be implemented within the curriculum (48). A focus should also be put on finding coping strategies and identifying vulnerable subgroups for interventions that need to be targeted in future pandemics (32).

## Conclusion

We found that a substantial proportion of university students in Germany suffered from worsened financial situation during the COVID-19 pandemic and their worsened financial situation was associated with higher levels of depressive symptoms. The worsened financial situation was caused to a great extent by loss of job possibilities due to contact restrictions. The typical students' jobs do not have securities of regular employment, and some students still have to rely on those for their living support. This shows that university students are a specific vulnerable group, which needs to be considered when pandemic control measures are implemented.

## Data Availability Statement

Due to the nature of this research, participants of this study did not agree for their data to be shared publicly, so supporting data are not publicly available. Data are available on request from the corresponding author for collaborating researchers within the C19 ISWS consortium, as consent for this was provided from all participants.

## Ethics Statement

The study was conducted according to the guidelines of the Declaration of Helsinki, and approved by the Ethics Committee of all five participating universities [Charité—Universitätsmedizin Berlin, University of Bremen (protocol code 2020-04-EILV, dated 4 May 2020), Heinrich-Heine University Duesseldorf (protocol code 2020-958, dated 5 May 2020), University of Siegen (protocol code ER 08/2020, dated 7 May 2020), and Martin-Luther University Halle-Wittenberg (protocol code 2020-066, dated 10 June 2020)]. The patients/participants provided their written informed consent to participate in this study.

## Author Contributions

SN, NK, and AS-P developed the study questions for this investigation. NK conducted the statistical analyses and prepared data visualizations. SN and NK jointly wrote the first draft of the manuscript. All authors contributed to conception and design, acquisition of data, and interpretation of the analyses, critically revised the content of the article and approved the submitted version.

## Funding

We acknowledge the financial support of the Open Access Publication Fund of the Martin-Luther-University Halle-Wittenberg. The funders had no role in study design, data collection and analysis, decision to publish, or preparation of the manuscript.

## Conflict of Interest

The authors declare that the research was conducted in the absence of any commercial or financial relationships that could be construed as a potential conflict of interest.

## Publisher's Note

All claims expressed in this article are solely those of the authors and do not necessarily represent those of their affiliated organizations, or those of the publisher, the editors and the reviewers. Any product that may be evaluated in this article, or claim that may be made by its manufacturer, is not guaranteed or endorsed by the publisher.
